# *Brucella abortus* infection exploits ZDHHC3-mediated STAT3 palmitoylation to regulate host responses and promote persistence

**DOI:** 10.1186/s13567-025-01681-y

**Published:** 2026-01-05

**Authors:** Xiaofeng Liu, Juntong Yu, Yanjie Chen, Haoran Liu, Ning Liu, Qisheng Peng

**Affiliations:** 1 State Key Laboratory for Diagnosis and Treatment of Severe Zoonotic Infectious Diseases, Key Laboratory for Zoonosis Research of the Ministry of Education, Institute of Zoonosis, College of Veterinary Medicine, Jilin University, Changchun, Jilin China; 2 Clinical Laboratory, Tumor Hospital of Jilin Province, Changchun, Jilin China; 3 Central Laboratory, The Second Hospital of Jilin University, Changchun, Jilin China

**Keywords:** *Brucella*, STAT3, ZDHHC, palmitoylation

## Abstract

*Brucella abortus*, an intracellular bacterium, employs intricate mechanisms to manipulate host signaling for persistence. This study investigates the role of STAT3 palmitoylation in *B. abortus*-infected macrophages. We demonstrate that *B. abortus* infection induces STAT3 palmitoylation, which is critical for its membrane recruitment. Among zinc finger DHHC-type palmitoyl acyltransferases (ZDHHC) family members, ZDHHC3 specifically mediates STAT3 palmitoylation in infected macrophages. ZDHHC3-induced STAT3 palmitoylation promotes STAT3 phosphorylation at Y705, independently of IL-6. Functionally, ZDHHC3 suppresses pro-inflammatory cytokines (IL-1β, TNF-α) and nitric oxide (NO) production, while increasing anti-inflammatory IL-10, thereby enhancing intracellular *B. abortus* survival. In vivo, ZDHHC3 mRNA is upregulated in splenic macrophages during infection, and 2-bromopalmitate (2BP) treatment reduces bacterial burden in mice, associated with elevated TNF-α and IFN-γ. Additionally, ZDHHC3 inhibits macrophage apoptosis (via regulating Bax and Bcl-2), limiting bacterial egress from apoptotic cells. These findings identify ZDHHC3-mediated STAT3 palmitoylation as one of the key regulatory mechanism in *B. abortus* infection, linking lipid modification to STAT3 activation, inflammation, apoptosis, and bacterial persistence.

**Supplementary Information:**

The online version contains supplementary material available at 10.1186/s13567-025-01681-y.

## Introduction

*Brucella abortus* is a facultative intracellular Gram-negative bacterium and the causative agent of brucellosis, a zoonotic disease characterized by chronic infections in humans and animals, leading to significant public health and economic burdens worldwide [1]. A key virulence strategy of *B. abortus* is its ability to survive and replicate within macrophages by manipulating host signaling pathways, particularly those involved in immune regulation and cell survival [2]. Among these pathways, the signal transducer and activator of transcription 3 (STAT3) plays a central role, as its activation modulates inflammatory responses, apoptosis, and antimicrobial defenses [3].

STAT3 siRNA treatment elevated the expression of proinflammatory cytokines, including IL-1α, IL-12, and TNFα in *Brucella*-infected macrophages [4]. Knock-down of STAT3 markedly can reduce the IL10-promoted *Brucella* persistence in RAW 264.7 cells [5]. However, the data from another lab demonstrated that inhibition of the STAT3 activity with AG490 during the stage of *Brucella* infection suppressed proinflammatory responses, apoptosis, and increased *Brucella* intracellular survival within macrophages [6]. Although these results are contradictory, these data show that STAT3 plays an important role in mediating *Brucella* infection in macrophages. However, if we want to decipher the exact role of STAT3 during *B. abortus* infection, the molecular mechanisms governing STAT3 regulation should be further investigated.

Post-translational modifications, such as phosphorylation, are well-known regulators of STAT3 activity, with phosphorylation at tyrosine 705 (Y705) being critical for its transcriptional function [7]. Membrane recruitment of STAT3 is a prerequisite for its phosphorylation [8], and given that *B. abortus* primarily resides within macrophages, understanding how STAT3 is localized and activated in these cells is critical for deciphering bacterial pathogenesis. Additionally, STAT3 activation is often linked to immunosuppression, which aligns with *B. abortus*’s ability to elicit minimal inflammatory responses [9]—a strategy that promotes chronic infection. Notably, interleukin-6 (IL-6) is a known inducer of STAT3 phosphorylation and has been implicated in maintaining *B. abortus* persistence in macrophages [4, 5, 10], but whether IL-6 intersects with other regulatory mechanisms of STAT3 during infection requires clarification.

More recently, palmitoylation—a reversible lipid modification involving the covalent attachment of palmitate to cysteine residues—has emerged as a key determinant of protein membrane localization and signaling potential [11]. Palmitoylation is catalyzed by zinc finger DHHC-type palmitoyl acyltransferases (ZDHHCs), a family of enzymes with distinct substrate specificities in different cellular contexts [11]. For STAT3, palmitoylation has been reported to regulate its membrane recruitment and activation in various cell types, with ZDHHC3, ZDHHC5, and ZDHHC7 identified as potential acyltransferases [7, 12]. However, whether STAT3 undergoes palmitoylation during *B. abortus* infection, and if so, which ZDHHC mediates this modification, remains unknown.

Against this backdrop, the present study aimed to investigate the role of palmitoylation in STAT3 regulation during *B. abortus* infection. Specifically, we sought to: (1) determine if *B. abortus* infection induces STAT3 palmitoylation and its impact on STAT3 membrane recruitment; (2) identify the ZDHHC acyltransferase responsible for STAT3 palmitoylation in infected macrophages; (3) examine the relationship between palmitoylation and phosphorylation of STAT3, including potential crosstalk with IL-6 signaling; and (4) evaluate the functional consequences of this pathway on inflammatory responses, apoptosis, and bacterial survival both in vitro and in vivo.

By addressing these questions, we aim to uncover novel molecular mechanisms by which *B. abortus* manipulates host cell signaling, potentially identifying therapeutic targets for combating brucellosis.

## Materials and methods

### Ethics statement

All animal experiments were conducted in accordance with the Guidelines for the Care and Use of Laboratory Animals of Jilin University and approved by the Institutional Animal Care and Use Committee (IACUC approval number: KT202408159).

### Antibodies and reagents

The following antibodies and reagents were purchased from biological companies. Antibodies were as follows: STAT3 (Cat# 9139), phospho-STAT3 (Y705, Cat# 9145, CST), Bax (Cat#5023, CST), β-actin (Cat#4970, CST), anti-rabbit IgG HRP (Cat#7074S, CST), anti-mouse IgG HRP, (Cat#7076S, CST), FLAG (Cat#14793, CST), ZDHHC3 (Cat# sc-377378, Santa Cruz Biotechnology), DHHC5 (Sigma, Cat# HPA014670, Sigma,), DHHC7 (Cat#ab138210, Abcam) and streptavidin-HRP(Cat#A0303, Beyotime). Reagents were as follow: 2-bromopalmitate (2BP, Cat#E0120, Selleckchem), dimethyl sulfoxide (DMSO, Cat# D2650, Sigma), Protein A/G agarose (Cat#sc-2003, Santa Cruz Biotechnology), streptavidin agarose (Cat#20359, Thermo Fisher Scientific), anti-Flag agarose gel (Cat#A2220, Sigma), RNA extraction kit (Cat#74104, Qiagen), reverse transcription kit (cat. no. Cat#330401, Qiagen), SYBR Green PCR Master Mix (Cat#4367659, Thermo Fisher Scientific), TUNEL assay kit (Cat# 11684795910, Roche Diagnostics), ELISA kits for mouse INFγ (Cat # PA5-97783, Thermo Fisher Scientific), TNF-α (Cat # BMS607-3, Thermo Fisher Scientific), IL-1β (Cat # BMS6002-2TEN, Thermo Fisher Scientific) and IL-10 (Cat # BMS614TEN, Thermo Fisher Scientific), IFN-γ (Cat #88-7314, Thermo Fisher Scientific).

### Generation of RAW264.7 derivative cell lines

The STAT3 gene from RAW264.7-derived cDNA was obtained using PCR (Forward primer: ATGGCTCAGTGGAACCAGCTGC, Reverse primer: TCACATGGGGGAGGTAGCACA), and then was inserted into pCDNA3.1 vector using ClonExpress MultiS One Step Cloning Kit (Cat #C113-01) according to manufacturer’s instructions. Flag epitopes were introduced by PCR-driven overlap extension. After the plasmid was verified by DNA sequencing, pCDNA3.1-FLAG-STAT3 vector was transfected into the RAW264.7 cell line using Escort^™^ IV Transfection Reagent ((Cat #L3287, Sigma), and stable lines were generated by selection with 400 μg/mL G418 (Invitrogen) [13].

RAW264.7 knockout cell lines (ΔZDHHC3, ΔZDHHC5, ΔZDHHC7, ΔIL-6) were generated using CRISPR/Cas9 technology as previously described [7, 12, 14, 15]. RAW-NT (non-targeted) cells served as controls. Briefly, two sgRNAs of ZDHHC3, ZDHHC5, ZDHHC7 or IL-6 were cloned into LentiCRISPRv2, respectively. And then RAW264.7 macrophages (5 × 10^5^/well) were transfected with two sgRNA constructs of ZDHHC3, ZDHHC5, ZDHHC7 or IL-6. On day 2, puromycin (1 µg/mL) was added into cells to screen stable colonies. Primers of sgRNA of ZDHHC3: sgRNA1: AACTCTGCCTCCACGAGGCA; sgRNA2: CCGACACAGTTGTTGACCCA. Primers of sgRNA of ZDHHC5: sgRNA1: CTGTCTCCACGACTCAACTG; sgRNA2: GTTGCAGTCCATTCGTTCAG. Primers of sgRNA of ZDHHC7: sgRNA1: GAGGATGATGCTCGACGTCC; sgRNA2: GGACGTCGAGCATCATCCTC. Primers of sgRNA of IL-6: sgRNA1: TCTGGAGTACCATAGCTACC; sgRNA2: TATACCACTTCACAAGTCGG.

### Separation of spleen macrophages

CD11b^+^ macrophages were isolated from the spleens of *B. abortus*-infected or uninfected C57BL/6 mice with a CD11b magnetic microbeads cell sorting kit Thermo Fisher Scientific (Cat#8802-6860-74) by following the manufacturer’s instructions, as previously described [16].

### Macrophage infection and survival assay

RAW264.7 cells (2.0 × 10^5^) were cultured in αMEM with 10% fetal bovine serum (FBS) at 37 °C with 5% CO_2_. And then, cells were infected with *B. abortus* at MOI (multiplicity of infection) of 100:1 by centrifuging *B. abortus* into cells at 400 g at 4 °C for 10 min. Following 15 min of infection in an atmosphere containing 5% CO_2_ at 37 °C, αMEM medium was used to wash the cells three times to take off extracellular *B. abortus* and infected for another 60 min in αMEM with 40 μg/mL gentamicin to kill extracellular Brucella. Thereafter, the antibiotic concentrations were decreased to 10 μg/mL [17]. To investigate *Brucella* intracellular growth, infected cells were lysed with PBS with 0.1% Triton X-100 at indicated time points, and the serial dilutions of lysates were plated immediately into TSA plates to count CFUs [17].

### Drug treatment

For inhibition of ZDHHC activity, cells were pretreated with 50 μM 2BP (dissolved in DMSO) or DMSO alone (vehicle control) for 2 h before *B. abortus* infection, and maintained in the presence of 2BP or DMSO during infection.

### Membrane fraction isolation

Membrane and cytosolic fractions were isolated using the Mem-PER Plus Membrane Protein Extraction Kit (Cat#89842, Thermo Fisher Scientific) according to the manufacturer’s instructions. Briefly, cells were lysed in permeabilization buffer, centrifuged to separate cytosolic (supernatant) and membrane (pellet) fractions, and membrane proteins were solubilized in solubilization buffer. Protein concentration was determined using the BCA Protein Assay Kit (Cat# 23225, Thermo Fisher Scientific).

### Acyl-biotin exchange (ABE) assay

The ABE assay was performed as previously described with minor modifications [18]. In brief, RAW264.7 cells expressing Flag-tagged STAT3 were washed with cold PBS. Prior to cell lysis, N ethylmaleimide (NEM) was dissolved in 100% EtOH and added to the Lysis Buffer (50 mM Tris-HCl pH 7.5, 150 mM NaCl, 1 mM MgCl2, 1% NP-40, 10% glycerol with phosphatase inhibitor cocktail (Cat#P0044, Sigma) and protease inhibitor cocktail (Cat#P8340, Sigma) to a final concentration of 50 mM. Cells were then suspended in NEM containing lysis buffer for 1.5 h at 4 ℃ and the supernatants were incubated with anti-Flag agarose beads (Cat#A2220, Sigma) at 4 ℃ for 3 h. After incubation, the beads were washed five times with Lysis Buffer of pH 7.5 and then three times with Lysis Buffer of pH 7.2. Then, beads were incubated with a freshly prepared hydroxylamine (HAM)-containing lysis buffer (50 mM Tris-HCl pH 7.2, 150 mM NaCl, 1 mM MgCl2, 1% NP-40, 10% glycerol, 1 mM HAM and protease inhibitor cocktail (Cat#P8340, Sigma) at room temperature for 1 h and washed four times with Lysis Buffer of pH 7.2 and three times with Lysis Buffer of pH 6.2. Subsequently, beads were treated with Biotin-BMCC (5 μM) in Lysis Buffer (pH 6.2) at 4 ℃ for 1 h. The immunoprecipitate samples were analyzed by western blot using anti- FLAG (Cat#14793, CST) and streptavidin-HRP (Cat#A0303, Beyotime).

### Co-immunoprecipitation (Co-IP)

RAW264.7 cells were lysed in IP buffer (50 mM Tris-HCl [pH 7.4], 150 mM NaCl, 1 mM EDTA, 1% NP-40, and protease (Cat#P8340, Sigma) (Cat#P0044, Sigma) and phosphatase inhibitors (Cat#P0044, Sigma)). Then incubated with anti-ZDHHC3 antibody overnight at 4 °C. Protein A/G agarose was added for 4 h, and beads were washed 5 times with IP buffer. Immunoprecipitated proteins were eluted with Laemmli buffer and analyzed by Western blot with anti-STAT3 antibody.

### Western blot analysis

Proteins of lysates (20–30 μg) were separated by SDS-PAGE and transferred to PVDF membranes (Cat#IPVH00010, Millipore). Membranes were blocked with 5% non-fat milk in TBST (20 mM Tris-HCl [pH 7.6], 150 mM NaCl, 0.1% Tween-20) for 1 h, incubated with primary antibodies overnight at 4 °C, followed by HRP-conjugated secondary antibodies for 1 h at room temperature. Bands were visualized using ECL Western Blotting Substrate (Cat#32106, Thermo Fisher Scientific).

### Real-time PCR (RT-PCR)

For each real-time reaction, reverse transcribed cDNA products (2 μL) were amplified by PCR in a total volume of 25 μL with 10 p moles each of related forward and reverse primer. Total RNA was extracted using RNA extraction kit (Cat#74104, Qiagen), reverse-transcribed into cDNA (cat. no. Cat#330401, Qiagen). Primers used were: ZDHHC3 (forward: GGGCCTGCTCTTCCTCATTT, reverse: AGCCTAGAGAAGGGGTGG) [19], Bcl-2 (forward: TTTCTCTCTTTCGGCCGTGG, reverse: GACATCTCCCTGTTGACGCT) [20], Bax (forward: CCAAGAAGCTGAGCGAGTGT, reverse: CCGGAGGAAGTCCAATGTC) [21], and GAPDH (forward: ATCCACGAAACTACCTTCAA, reverse: ATCCACACGGAGTACTTGC, internal control) [21]. The fold change of mRNA levels in cells was obtained through the comparative threshold cycle (Ct) method [13].

### Determination of NO production

Supernatant of each cell sample was collected at certain times point after B. abortus challenge. Nitric oxide (NO) content was measured by analyzing its stable product, nitrite, using a Griess reagent (Cat#215-981-2 Sigma) as previously described [13]. Data are expressed as micromoles of nitrite (mean ± SEM).

### Measurements of cytokine in culture supernatants

Culture supernatants or splenocyte supernatants were collected, and IL-1β (Cat # BMS6002-2TEN, Thermo Fisher Scientific), TNF-α (Cat # BMS607-3, Thermo Fisher Scientific), IL-10(Cat # BMS614TEN, 88-7105 Thermo Fisher Scientific), IFN-γ (Cat #88-7314, Thermo Fisher Scientific) levels were measured using ELISA kits according to the manufacturer’s instructions.

### TUNEL assay

Apoptosis was detected using the TUNEL assay kit (Cat# 11684795910, Roche Diagnostics) according to the manufacturer’s instructions. Briefly, cells were fixed with 4% paraformaldehyde, permeabilized with 0.1% Triton X-100, incubated with TUNEL reaction mixture for 1 h at 37 °C, and counterstained with DAPI. TUNEL-positive cells were visualized under a fluorescence microscope (Zeiss) and quantified as the percentage of total cells.

### Animal experiments

C57BL/6 mice (6–8 weeks old, male) were purchased from Laboratory Animal Center of Jilin University. Mice were intraperitoneally injected with 2BP (50 mg/kg) [22] or PBS (control) daily for 3 days before infection and throughout the experiment. Mice were infected intraperitoneally with 1 × 10^6^ CFU of *B. abortus*. To determine the early phase of *B. abortus* infection in Vivo, mice were euthanized at 1-week post-infection, spleens were weighed, and homogenized to count bacterial CFU on *Brucella* agar plates. For splenocyte stimulation, spleen cells were isolated, seeded at 2 × 10⁶ cells/well, and stimulated with *B. abortus* (MOI 100), 5 μg/mL ConA (Cat#11028-71-0, Sigma), or 1 μg/mL *Escherichia coli* LPS (Cat#93572-42-0, Sigma) for 48 h. Supernatants were collected for TNF-α or IFN-γ detection by ELISA.

### Statistical analysis

All values were expressed as means ± SD. Data were analyzed using a one-way ANOVA followed by Bonferroni correction. For statistical comparison between two groups, we used an unpaired a Newman-Student t test. All statistical analysis was performed with SPSS 10.0 software. A two-sided *p* value < 0.05 was considered to be significant [13].

## Results

### *B. abortus* infection promotes STAT3 palmitoylation

**Figure 1:**
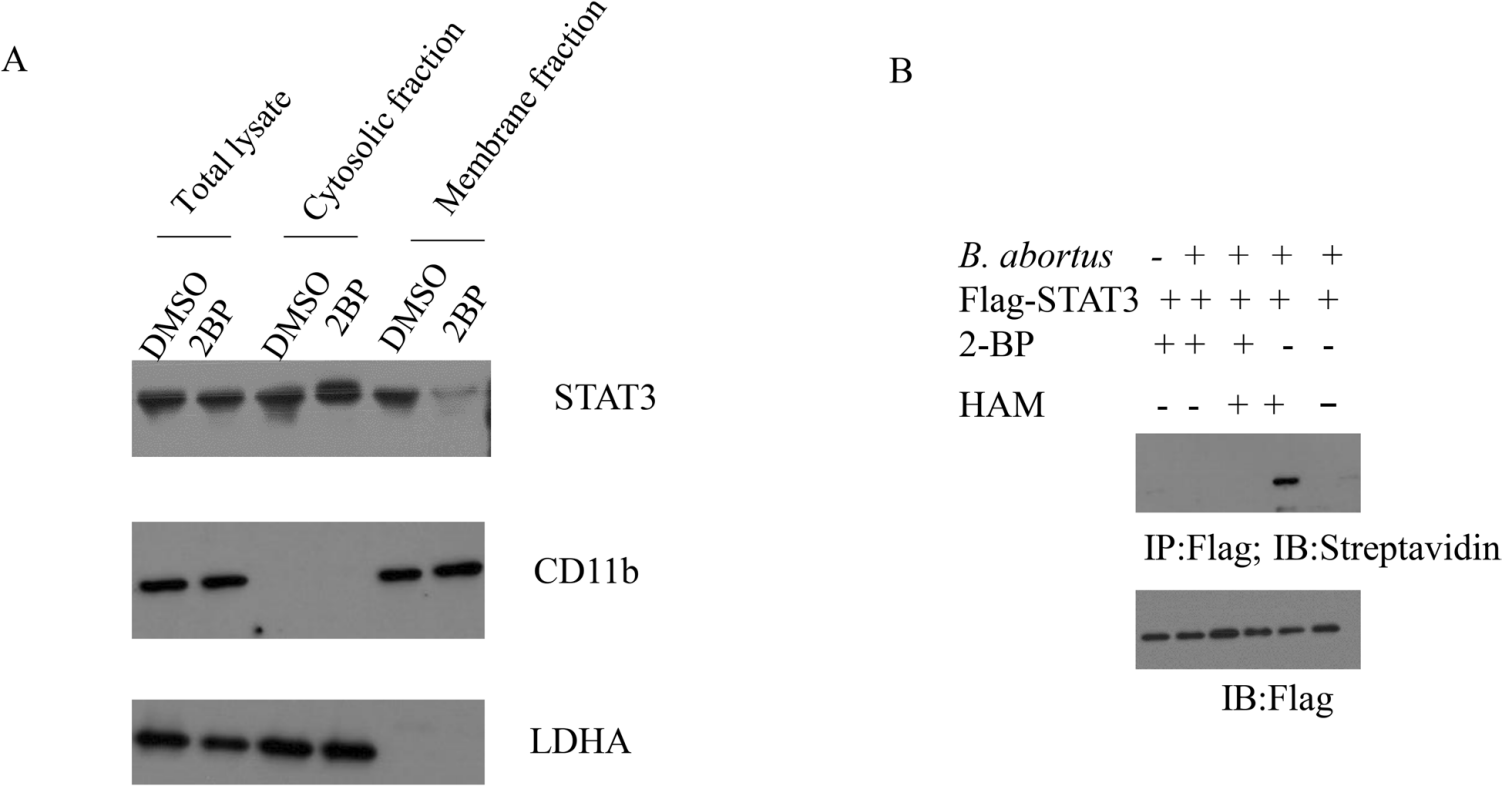
***B. abortus*** **infection promotes STAT3 palmitoylation**. **A** Subcellular fractionation of STAT3 was performed in the *B. abortus*-infected RAW264.7 cells in the presence of 2BP (100 μM) or DMSO. LDHA was designed as a cytosol marker). CD11b was designed as a membrane marker. **B** RAW264.7 cells that expressed Flag–STAT3 were infected with *B. abortus*. Palmitoylation levels of STAT3 were detected using Acyl-Biotin Exchange (ABE) assay in the presence of 2BP (100 μM) or HAM. The above data are representative of two independent experiments.

Recruitment of STAT3 to cell membrane plays a key role in its phosphorylation [8]. Firstly, we investigated whether palmitoylation would contribute to plasma membrane recruitment of STAT3 during the stage of *B. abortus* infection. Compared to DMSO treatment, 2-bromopalmitate (2BP), an inhibitor of the ZDHHC, treatment of RAW264.7 macrophages obviously decreased the levels of STAT3 in the membrane fraction but did not affect total STAT3 protein levels (Figure 1A). To visualize STAT3 palmitoylation in *B. abortus*-infected macrophages, the acyl-biotin exchange (ABE) assay was performed. Overexpressed STAT3 was palmitoylated, and its palmitoylation level failed to occur upon treatment with 2BP. Loss of signal in the absence of hydroxylamine (HAM) suggested that STAT3 incorporates palmitate through a thioester linkage (Figure 1B). These data demonstrate that STAT3 is palmitoylated in response to *B. abortus* infection.

### The palmitoyl acyltransferase ZDHHC3 regulates STAT3 palmitoylation

**Figure 2:**
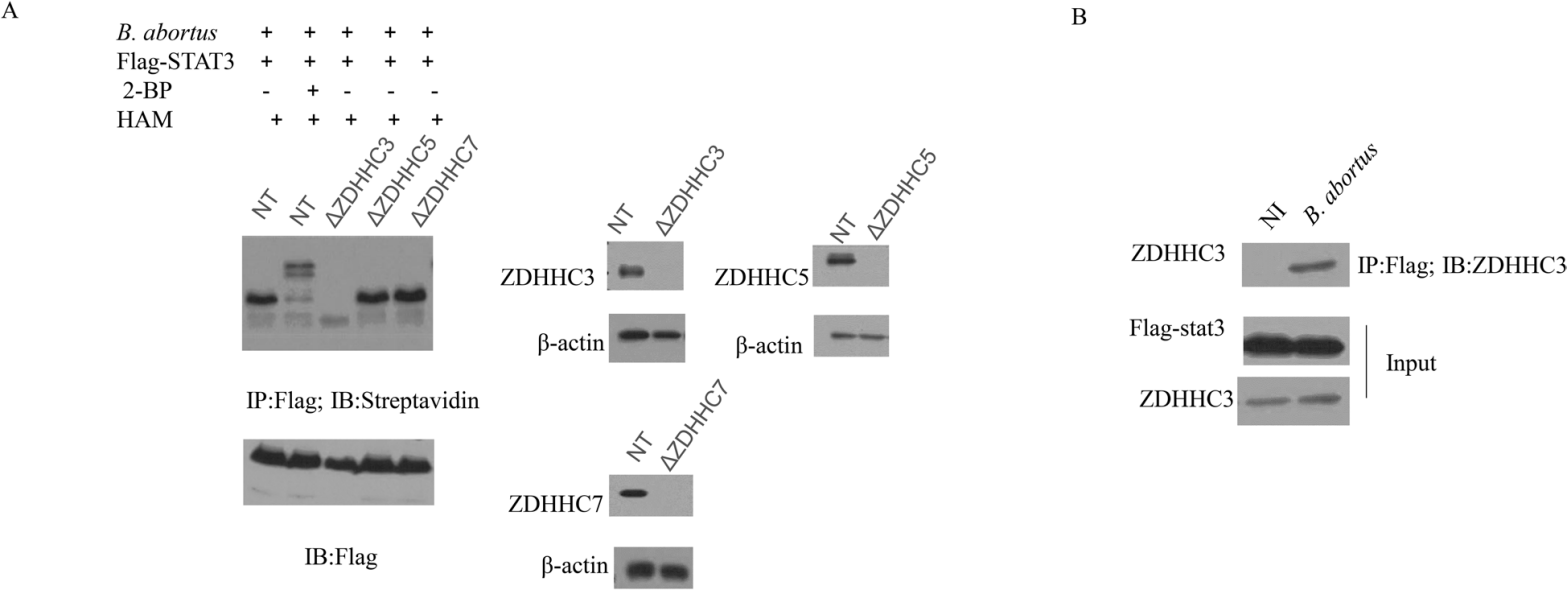
**The palmitoyl acyltransferase ZDHHC3 regulates STAT3 palmitoylation**. **A** RAW-NT, RAW-ΔZDHHC3, RAW-ΔZDHHC5, and RAW-ΔZDHHC7 macrophages that expressed Flag-STAT3 were infected with *B. abortus* in the presence of 2BP (100 μM) or HAM. STAT3 palmitoylation levels were detected using ABE assay. **B** Co-immunoprecipitation (Co-IP) assay was performed to check the interaction between ZDHHC3 and STAT3. Whole cell lysates were analyzed by anti-Flag immunoprecipitation followed by immunoblotting using the indicated antibodies. The above data are representative of two independent experiments. Non-infected RAW means NI.

Palmitoylation is catalyzed by zinc finger DHHC-type palmitoyl acyltransferases (ZDHHCs) [11]. Previous literatures indicated that STAT3 could be palmitoylated by ZDHHC3, ZDHHC5 or ZDHHC7 in different cells [7, 12]. Next, we examined which palmitoyl acyltransferase is responsible for STAT3 palmitoylation in *B. abortus*-infected macrophages. We used the ABE assay to analyze the levels of STAT3 palmitoylation in RAW.264.7 cells with deletion of ZDHHC3 (ΔZDHHC3), ZDHHC5 (ΔZDHHC5) or ZDHHC7 (ΔZDHHC7). Deletion of ZDHHC3 obviously reduced the level of STAT3 palmitoylation, whereas deletion of ZDHHC5 or ZDHHC7 had no effect on STAT3 palmitoylation compared to RAW-non-targeted (NT) control macrophages (Figure 2A). We therefore speculated that ZDHHC3 catalyzes STAT3 palmitoylation in *Brucella*-infected macrophages. To confirm our hypotheses, co-immunoprecipitation assays were performed to test the interaction between STAT3 and ZDHHC3. In RAW264.7 cells, *Brucella* infection induced the association of STAT3 and ZDHHC3, compared to non-infected cells (NI) (Figure 2B). Taken together, these data demonstrate that *Brucella* infection induces STAT3 palmitoylation by ZDHHC3 in macrophages.

### ZDHHC3-induced STAT3 palmitoylation during the stage of *B. abortus* infection mediates STAT3 phosphorylation

**Figure 3:**
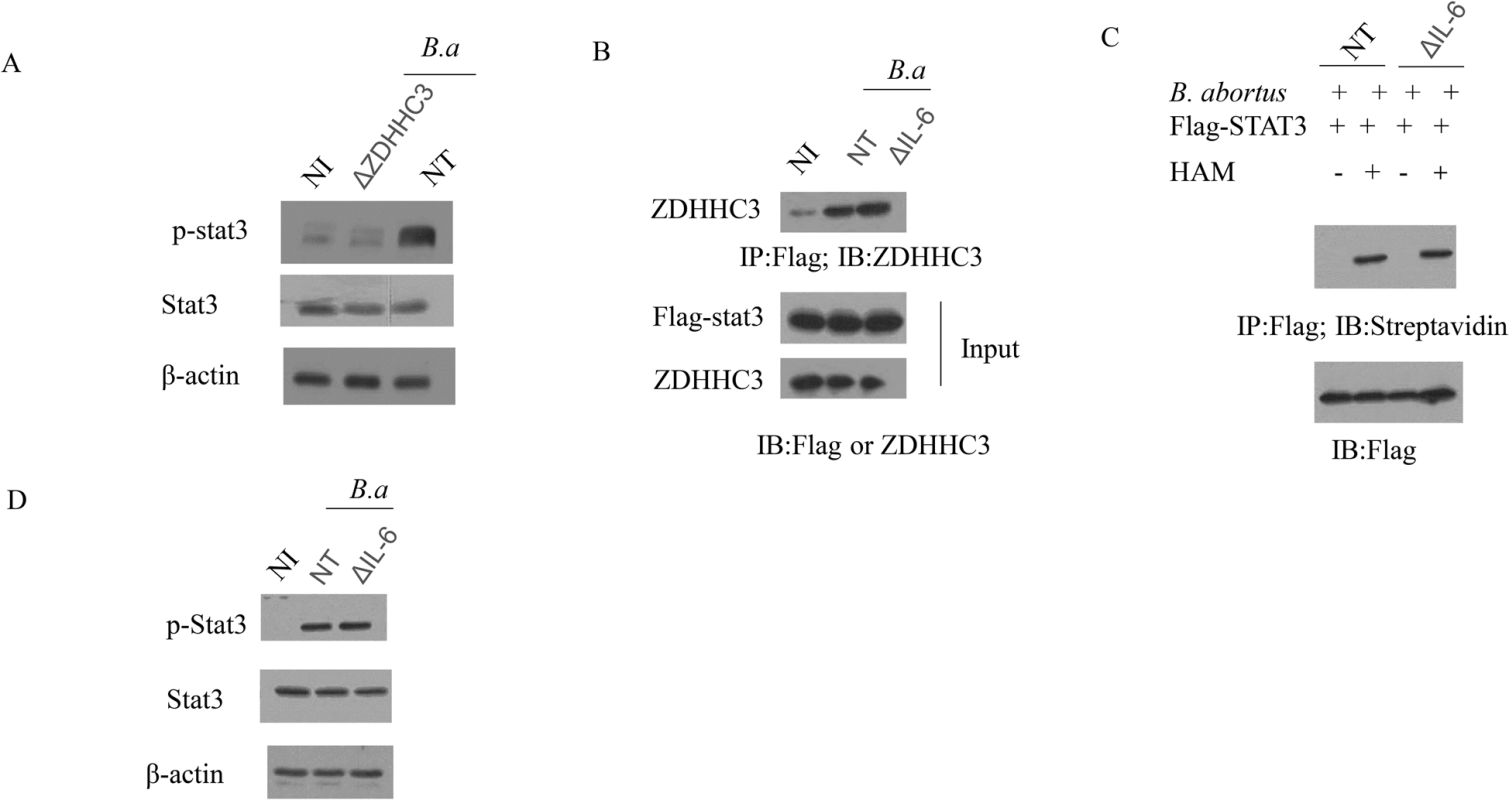
**ZDHHC3-induced STAT3 palmitoylation during the stage of** ***B. abortus*** **infection mediates STAT3 phosphorylation**. **A** The phosphorylation of STAT3 was detected in the NI, *B. abortus*-infected RAW-NT or RAW-ΔZDHHC3 macrophages. **B** RAW-NT and RAW-ΔIL-6macrophages that expressed Flag-STAT3 were non-infected (NI) or infected with *B. abortus.* Co-IP assay was performed to check the interaction between ZDHHC3 and STAT3. Whole cell lysates were analyzed by anti-Flag immunoprecipitation followed by immunoblotting using the indicated antibodies. **C** STAT3 palmitoylation levels were detected in the *B. abortus*-infected RAW-NT or RAW-IL-6 macrophages by using ABE assay. **D** The phosphorylation of STAT3 was detected in the NI, *B. abortus*-infected RAW-NT or RAW-IL-6 macrophages. The above data are representative of two independent experiments.

STAT3 phosphorylation at Y705 mediates its function [7]. Next, we investigated whether palmitoylation could promote STAT3 phosphorylation. As shown in Figure 3A, STAT3 phosphorylation was notably decreased by the deletion of ZDHHC3, compared to infected RAW-NT cells. IL-6 is known to induce STAT3 phosphorylation, and plays an important role in retaining *Brucella* infection in macrophages [4, 10]. Therefore, we deleted IL-6 in RAW cells (ΔIL-6) to determine if IL-6 was required for STAT3 palmitoylation. Co-immunoprecipitation assays showed that deletion of IL-6 did not affect *Brucella* infection-induced association of STAT3 and ZDHHC3 (Figure 3B). To confirm that IL-6 is not involved in STAT3 palmitoylation, STAT3 palmitoylation was measured by ABE assay in RAW-NT and ΔIL-6 cells in the presence or absence of HAM. The level of STAT3 palmitoylation was similar in ΔIL-6 macrophages and RAW-NT macrophages (Figure 3C). Consistent with the STAT3 palmitoylation, deletion of IL-6 did not affect STAT3 phosphorylation (Figure 3D). Taking the above data together, these results clearly demonstrate that STAT3 palmitoylation by ZDHHC3 promotes the membrane recruitment and phosphorylation of STAT3 during the stage of *B. abortus* infection in macrophages.

### ZDHHC3 suppresses inflammatory cytokine and NO production in*** Brucella***-infected macrophages

**Figure 4:**
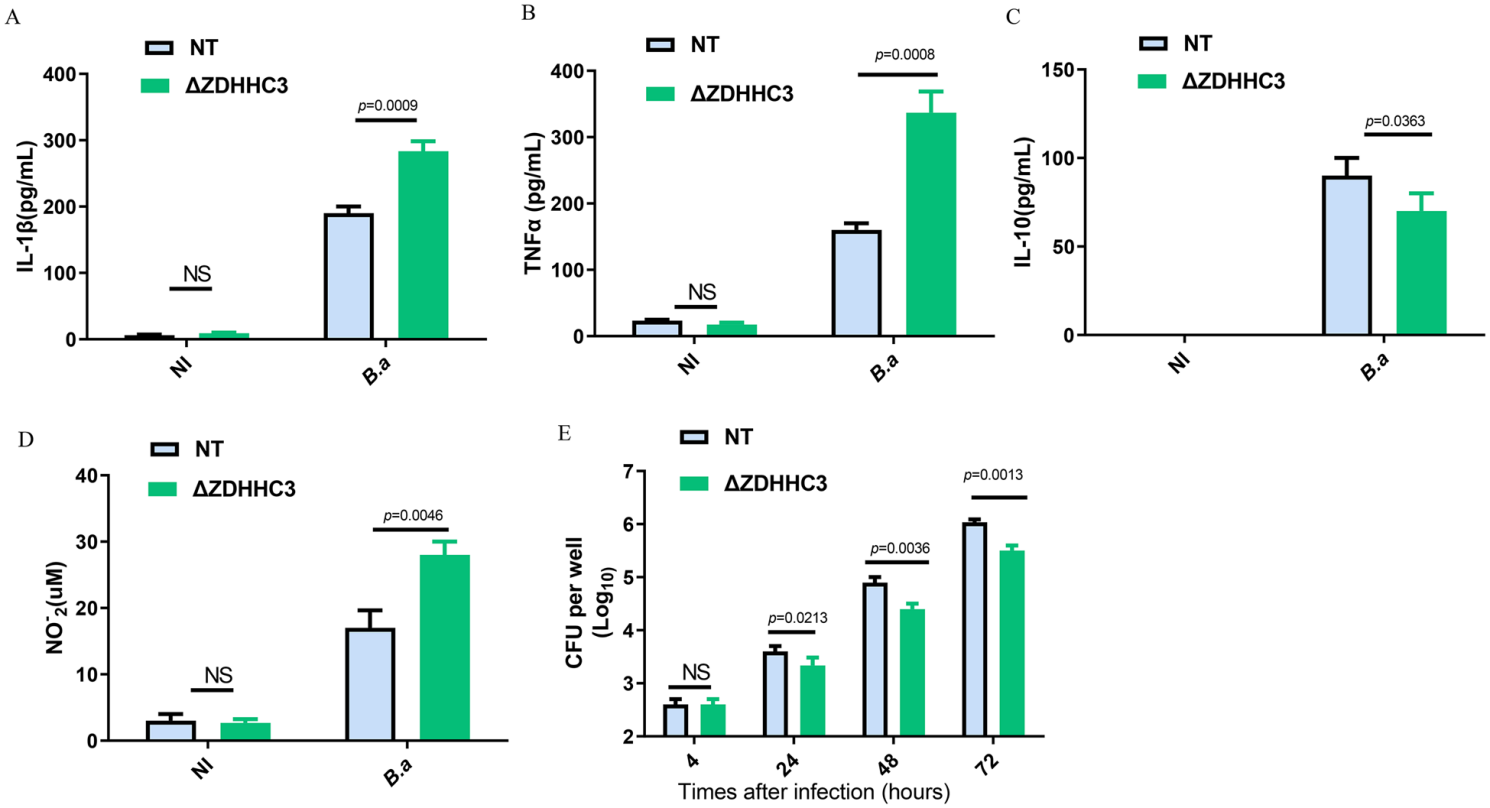
**ZDHHC3 suppresses inflammatory cytokine and NO production in *Brucella*-infected macrophages**. **A** RAW-NT and RAW-ΔZDHHC3 macrophages were infected or non-infected(NI) with *B. abortus* for 48 h. IL-1β (**A**), TNF-α (**B**) and IL-10 (**C**) in RAW-NT and RAW-ΔZDHHC3 macrophages media were detected using ELISA. **D** NO_2_^−^ (nitrite) accumulation in the media of macrophages was measured by Griess reaction. **E** RAW-NT and RAW-ΔZDHHC3 macrophages were infected with **B. abortus** for 4, 24, 48, or 72 h. *B. abortus* intracellular growth was measured using CFU assay. The data **A**–**D** are representative of three independent experiments. A one-way ANOVA followed by Newman-Student's t-test was used to determine the *P* value in (**A**–**E**). NS indicates no significance.

*Brucella* infection leads to minimal inflammatory responses in macrophages [9]. And phosphorylated STAT3 can cause immunosuppression. Therefore, we next investigate whether ZDHHC3 is involved in production of inflammatory cytokines. Our results demonstrated that the deletion of ZDHHC3 increased IL-1β release (Figure 4A) and TNF-α secretion in response to *Brucella* infection (Figure 4B). In contrast, deletion of ZDHHC3 reduced the production of the anti-inflammatory cytokine IL-10 (Figure. 4C), indicating that ZDHHC3 plays a role in inhibiting an inflammatory response during the stage of *Brucella* infection. Meanwhile, given that NO is an important marker of the M1 inflammatory macrophages [23], moreover, the production of NO is associated with the control of *Brucella* infection [17]. NO production was also evaluated in *Brucella*-infected RAW cells with and without ZDHHC3. Deletion of ZDHHC3 resulted in an increased NO production in comparison with *Brucella*-infected RAW-NT cells (Figure 4D). Consistent with NO production regulated by ZDHHC3, the absence of ZDHHC3 significantly decreased the survival of intracellular *Brucella* within macrophages (Figure 4E).

### ZDHHC3 is required for control of ***Brucella*** infection in vivo

**Figure 5:**
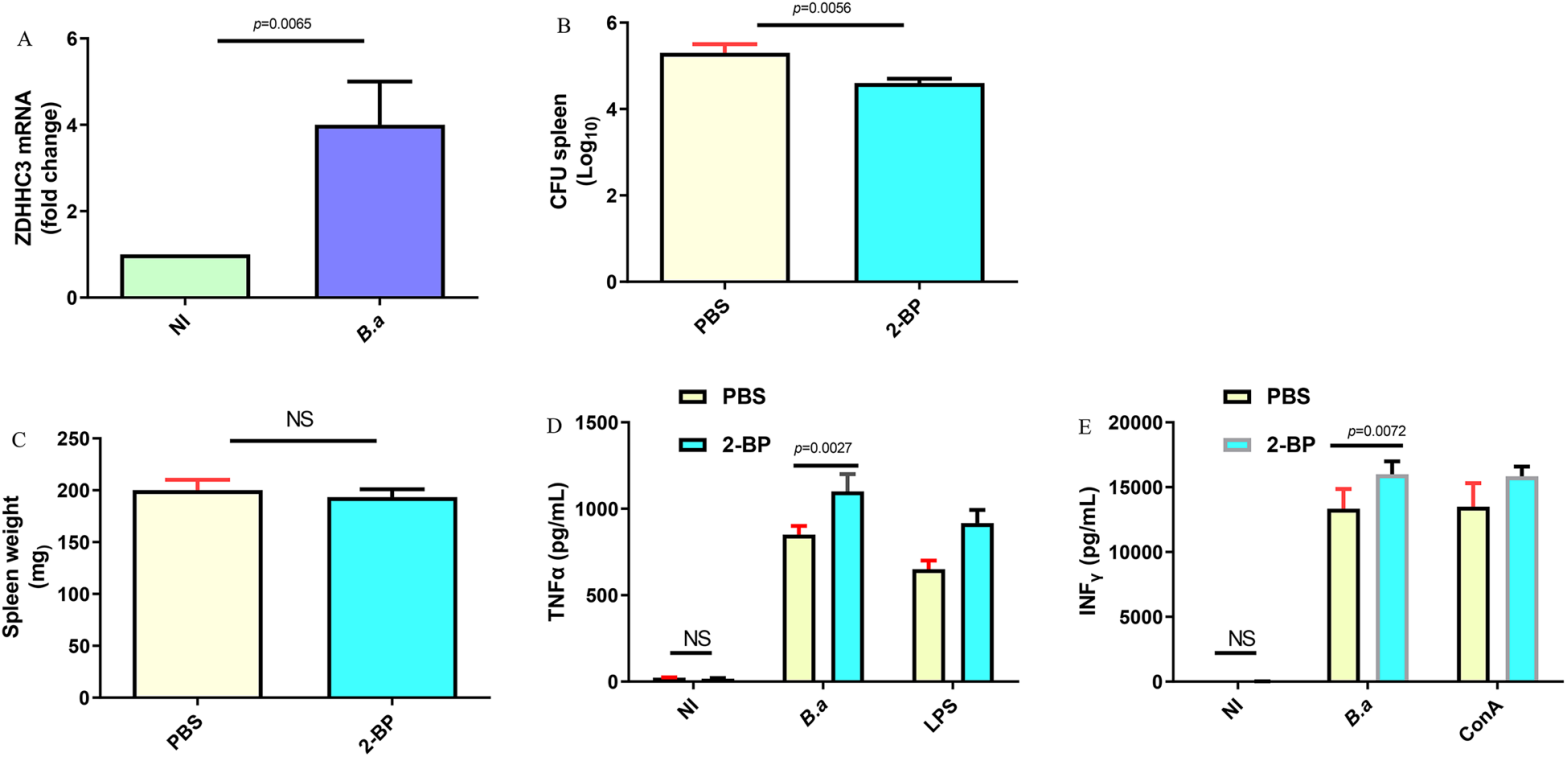
**ZDHHC3 is required for control of *Brucella* infection in vivo**. **A** Mice were infected intraperitoneally with 1 × 10^6^ CFU of *B. abortus* for 1 week, ZDHHC3 mRNA level was measured by RT-PCR in spleen CD11b macrophages. **B** Mice, which were treated with PBS or 2-BP, were infected intraperitoneally with 1 × 10^6^ CFU of *B. abortus* for 1 week. *B. abortus* growth within spleen was measured using CFU assay. **C** Spleen weight (mg) of LPS and 2-BP-treated mice were determined at 1 week. Splenocytes from 1 week-infected mice, which were treated with PBS or 2-BP, were stimulated with *B. abortus* for 48 h. 1 μg/mL LPS or 5 μg/mL ConA stimulation was designed as positive control. Nontreatment (NI) act as a negative control. TNFα (**D**) and IFNγ (**E**) in splenocyte media were detected using ELISA. The data are representative of three independent experiments. A one-way ANOVA followed by Newman-Student’s t-test was used to determine the *P* value in (**A**–**E**). NS indicates no significance.

Thus far, these data demonstrate that ZDHHC3 deletion in macrophages lead to increased production of inflammatory cytokines and reduced intracellular *Brucella*. Next, we examine the role of ZDHHC3 in host resistance against *Brucella* infection in vivo. Firstly, we investigated whether expression levels of ZDHHC3 is related to *Brucella* infection. CD11b macrophages from spleen were extracted and analyzed for ZDHHC3 mRNA. ZDHHC3 mRNA was upregulated in the spleen macrophages from in *Brucella* infected mice compared to non-infected mice (Figure 5A). To test if ZDHHC inhibitor would improve clearance of *Brucella*, infected mice were treated with PBS or 2-BP (an inhibitor of the ZDHHC) for 1 week. 2-BP treated mice had significantly decreased *Brucella* splenic colony forming units (CFU) compared to PBS treated mice (Figure 5B). Spleen weights were similar between PBS or 2-BP treated mice (Figure 5C).

As the production of IFNγ and TNFα is associated with the control of *Brucella* infection [24], to further confirm the relevance of ZDHHC3 in vivo, we examined whether the enhanced susceptibility of 2-BP treated mice at the stage of *Brucella* infection was correlated with IFNγ and TNFα secretion. Splenocytes from PBS or 2-BP treated mice, which were infected with *B. abortus* for 1 week, were stimulated with *B. abortus*. ConA or LPS stimulation was designed as positive controls. 2-BP treated mice produced higher levels of TNFα (Figure 5D) and IFNγ (Figure 5E) compared to PBS treated mice. Moreover, stimulation with LPS or ConAs can lead to TNFα and IFNγ secretion, respectively, suggesting the specificity of the response. Together, these data show that ZDHHC3 is required for control of *B*rucella infection in vivo.

### ZDHHC3 is involved in apoptosis caused by ***B. abortus*** infection

**Figure 6:**
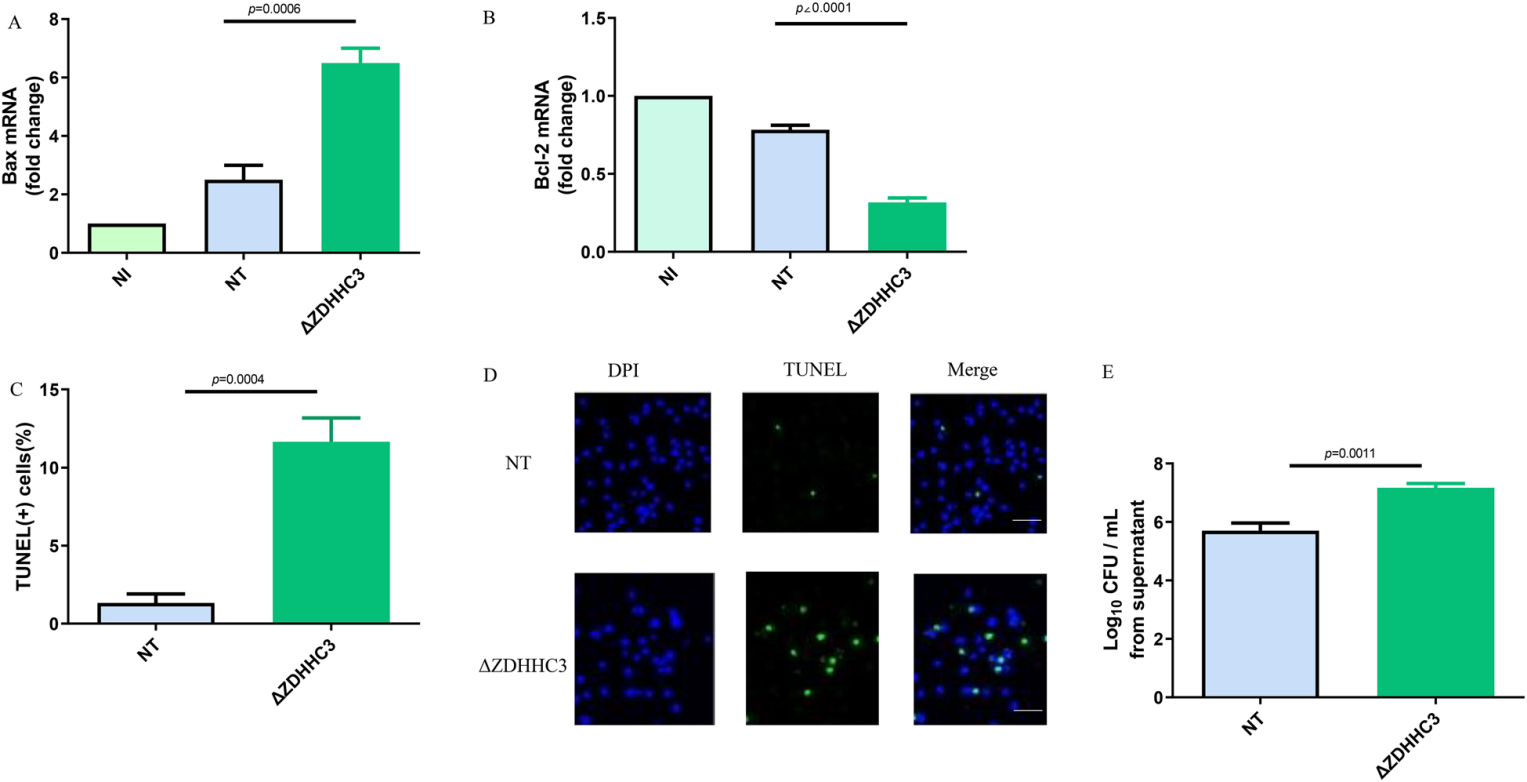
**ZDHHC3 is involved in apoptosis caused by *B. abortus*** **infection**. RAW-NT and RAW-ΔZDHHC3 macrophages were infected or non-infected (NI) with *B. abortus* for 48 h. Bax (**A**) and Bcl-2 (**B**) mRNA levels were measured by RT-PCR. **C** Apoptosis was detected by TUNEL assay. **D** The representative image of TUNEL staining. The data are representative of three independent experiments (The scale is 10 μm). **E** CFU assay expressing Log (CFU/mL) from the supernatant of RAW-NT and RAW-ΔZDHHC3 macrophages which were infected with *B. abortus* for 24 h, then incubated under reinfection‐permissive conditions for 24 h. A one-way ANOVA followed by Newman-Student’s t-test was used to determine the *P* value in (**A**–**C** and **E**). NS indicates no significance.

Intracellular *Brucella* can escape host killing via inhibiting macrophages apoptosis [25] and activated STAT3 can suppress apoptosis in cancer model [26]. Therefore, we investigated the role of ZDHHC3 in mediating macrophages apoptosis caused by *Brucella* infection by evaluating the expression of Bcl-2 and Bax, which play important role in regulating apoptosis [27]. RT- PCR (real-time polymerase chain reaction) was used to evaluate Bcl-2 and Bax mRNA levels in *Brucella*-infected macrophages. As shown in Figure 6A, *Brucella* infection induced significantly higher Bax mRNA levels in ΔZDHHC3 macrophages than in the RAW-NT or non-infected macrophages. In contrast, *Brucella* infection significantly suppressed Bcl-2 mRNA level in ΔZDHHC3 macrophages compared to the RAW-NT or non-infected macrophages (Figure 6B). These data suggest that ZDHHC3 may suppress macrophages apoptosis triggered by *Brucella* infection. To confirm the relevance of ZDHHC3 in mediating apoptosis, TUNEL assay was used to detect apoptotic cells. The data from Figures 6C, D clearly demonstrate that deletion of ZDHHC3 significantly promotes macrophages apoptosis induced by *Brucella* infection. In line with these data, and in the same reinfection‐permissive conditions, the number of *Brucella* in the supernatant at 48 h post infection was significantly increased in ΔZDHHC3 infected macrophages compared to RAW-NT macrophages (Figure 6E). This data suggests that bacterial egress from ΔZDHHC3 infected macrophages, in which apoptosis happens.

## Discussion

*Brucella abortus*, an intracellular Gram-negative bacterium, has evolved sophisticated strategies to manipulate host signaling pathways for its survival and replication within macrophages. In this study, we unravel a novel regulatory axis involving STAT3 palmitoylation, catalyzed by the palmitoyl acyltransferase ZDHHC3, which orchestrates multiple host responses during *B. abortus* infection.

The findings of this study advance our understanding of *Brucella* pathogenesis by integrating palmitoylation—an understudied post-translational modification (PTM)—into the pathogen’s immune evasion strategy. Prior work has focused on STAT3 phosphorylation [4–6] or other PTMs (e.g., ubiquitination) in *Brucella* infection [28], but palmitoylation’s role remained unknown. Our data align with broader trends in microbial pathogenesis, where pathogens increasingly exploit lipid modifications to manipulate host signaling: for example, *Salmonella* and *Shigella* target palmitoylation of NOD1/2 to modulate inflammasome activation [18], and *Mycobacterium tuberculosis* alters palmitoylation of immune checkpoints to enhance macrophages responses [29].

Notably, our identification of ZDHHC3 as a key mediator contrasts with studies in other systems. For instance, ZDHHC5 mediates STAT3 palmitoylation in oligodendrocytes [12], and ZDHHC7 regulates STAT3 in T helper 17 (Tₕ17) cells [7]. This discrepancy underscores that ZDHHC enzymes exhibit tissue- and cell-type-specific substrate preferences, likely driven by differences in co-chaperones, subcellular localization, or pathogen-induced remodeling of the host cell environment. For *B. abortus*, which primarily infects macrophages, ZDHHC3 emerges as the dominant acyltransferase for STAT3—a specificity that may reflect evolutionary adaptation of the pathogen to its primary host cell.

We established that ZDHHC3-induced STAT3 palmitoylation drives STAT3 phosphorylation at tyrosine 705 (Y705)—a modification critical for its transcriptional function. ΔZDHHC3 macrophages exhibited significantly reduced STAT3 Y705 phosphorylation compared to non-targeted (NT) controls, confirming palmitoylation as an upstream regulator of STAT3 activation. Notably, this phosphorylation was independent of interleukin-6 (IL-6), a canonical inducer of STAT3 signaling [8]. Deletion of IL-6 (ΔIL-6) had no impact on ZDHHC3-STAT3 interaction, STAT3 palmitoylation, or STAT3 phosphorylation. Our observation that STAT3 palmitoylation is IL-6-independent clarifies conflicting reports. Hop et al. showed that IL-6 promotes *B. abortus* clearance by enhancing macrophage bactericidal activity [4], while Erika et al. found that IL-6 is not involved in the control of bacterial replication in macrophages [24]. Our data resolve these by showing that *B. abortus* uses ZDHHC3 to activate STAT3 independently of IL-6, allowing it to decouple STAT3-mediated immunosuppression from IL-6 effects. This strategy is likely advantageous: IL-6 is often induced early in infection to recruit immune cells [24], but *B. abortus* can bypass this to activate STAT3 later, when dampening inflammation becomes critical for persistence.

Previous data demonstrated that inhibition of the JAK2/STAT3 pathway with AG490 significantly suppressed proinflammatory responses, apoptosis, and increased *Brucella* intracellular survival within macrophages, suggesting that the inhibition of STAT3 activation leads to bacterial replication [6]. Here, we showed that STAT3 activation is required for bacterial replication, because deletion of ZDHHC3 reduces bacterial replication in macrophages. The discrepancy between these results might be associated with the use of attenuated *Brucella* vaccine strain M5-90 for infection in the previous study, since independent study showed M5-90 infection has different inflammatory, apoptosis, and transport pathways compared to *B. abortus* [30]. In accordance with our data, it was previously reported that knock-down of STAT3 increases proinflammatory cytokines and decreases *B. abortus* persistence in macrophages [4, 5].

Despite its strengths, this study has limitation that warrant consideration. The mechanism by which *B. abortus* induces ZDHHC3-STAT3 interaction remains unclear. Does the pathogen secrete effectors that directly bind ZDHHC3 or STAT3? For example, *B. abortus* uses type IV secretion system (T4SS) effectors (e.g., BspI) to modulate host signaling [16]; future work should test whether T4SS effectors regulate ZDHHC3 activity or localization.

In summary, this study identifies ZDHHC3-mediated STAT3 palmitoylation as a central mechanism by which *B. abortus* subverts host immunity. By promoting STAT3 membrane recruitment and phosphorylation (independently of IL-6), ZDHHC3 suppresses inflammation, inhibits apoptosis, and enhances bacterial persistence. These findings not only advance our understanding of *Brucella* pathogenesis but also highlight palmitoylation as a therapeutic target for combating intracellular infections. Future work to dissect the molecular details of ZDHHC3 regulation and validate ZDHHC3-specific inhibitors will be critical for translating these insights into clinical solutions.

## Supplementary Information

**Additional file 1**

## Data Availability

The data that support the findings of this study are available from the corresponding author upon reasonable request.
