# Supplementary material for: Brucella abortus infection exploits ZDHHC3-mediated STAT3 palmitoylation to regulate host responses and promote persistence
Source: Vet Res. 2026 Jan 5;57:2. doi: 10.1186/s13567-025-01681-y (PMC12771712; doi:10.1186/s13567-025-01681-y)

Figure 1A Top panel

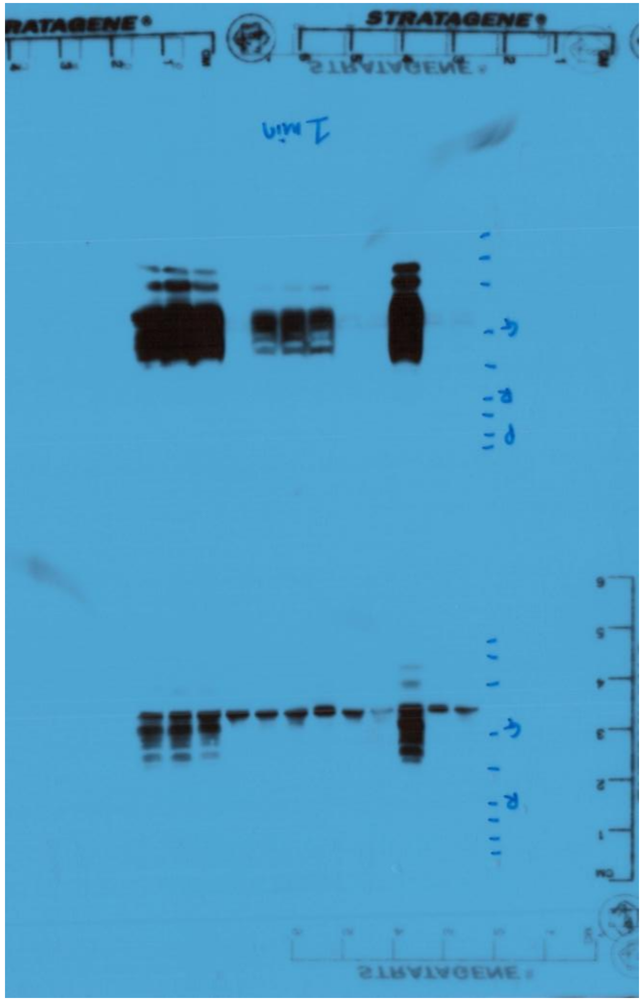

Figure 1A Middle panel

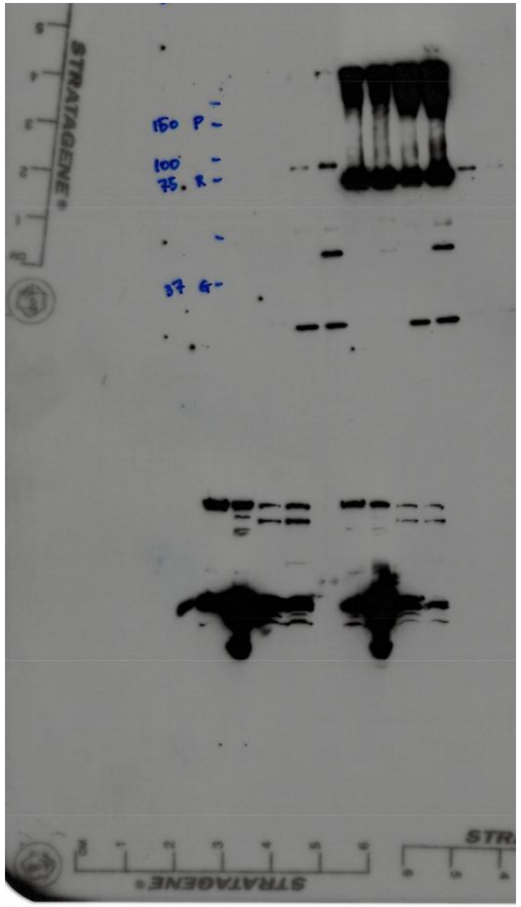

Figure 1A Bottom panel

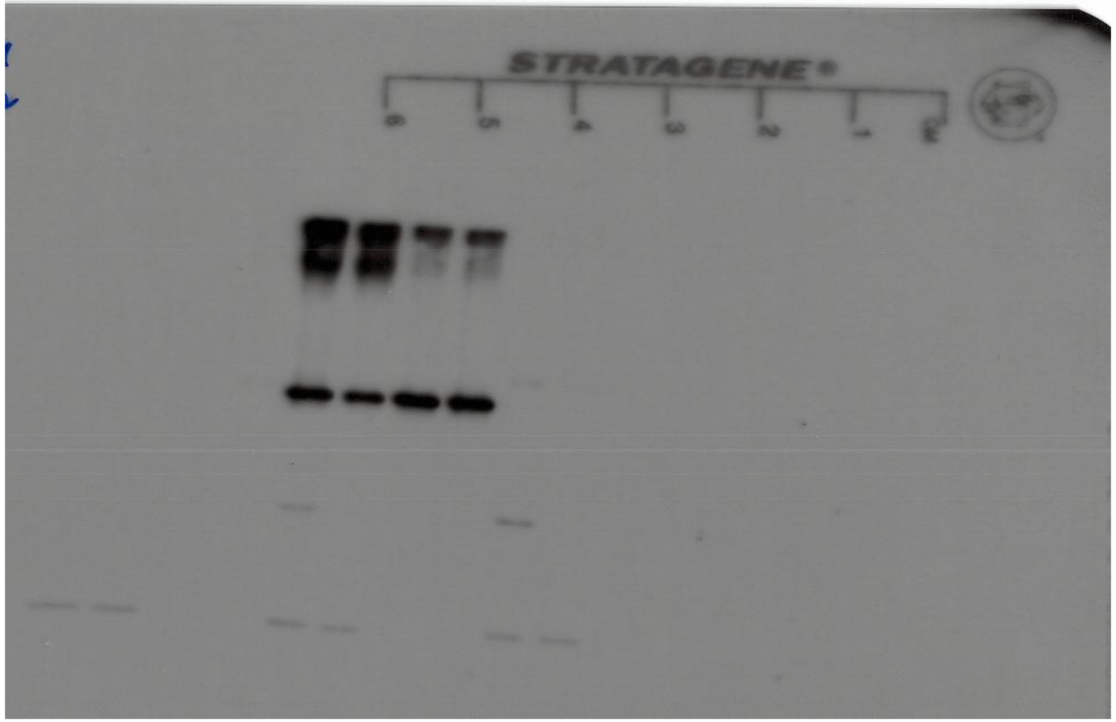

Figure 1B Top panel

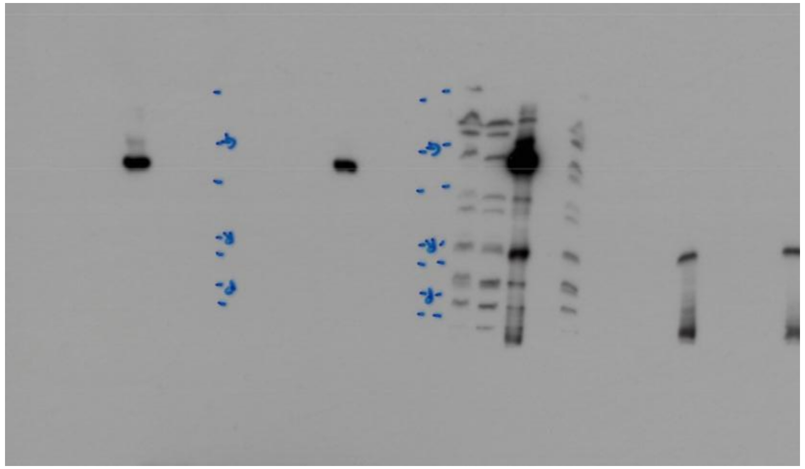

Figure 1B Bottom panel

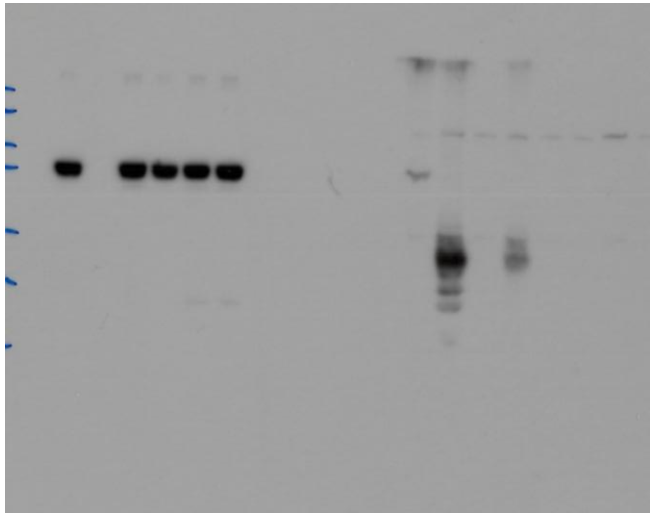

Figure 2A Top panel

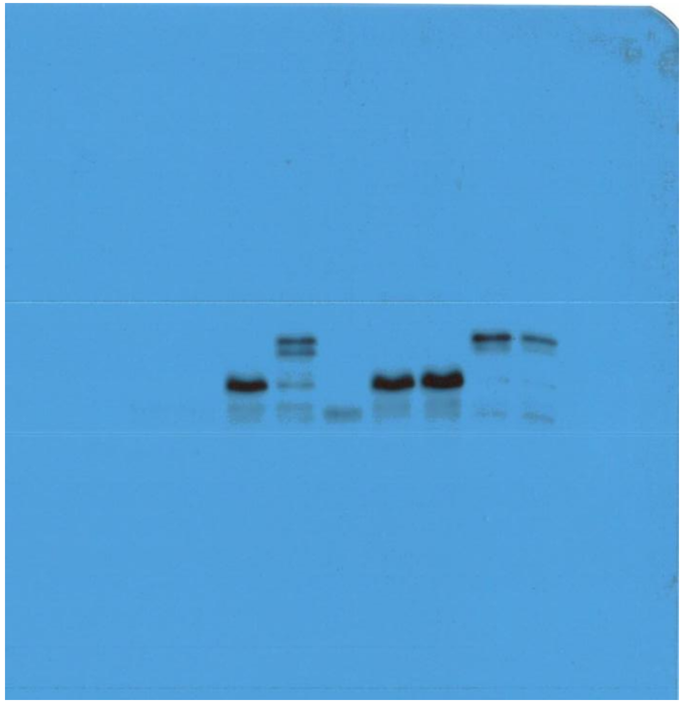

Figure 2 A bottom panel

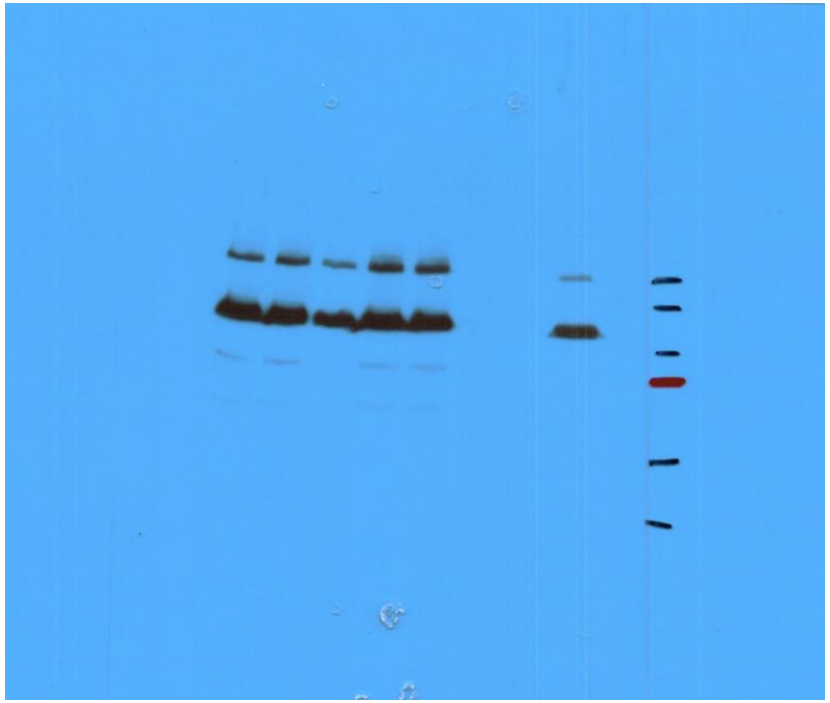

Figure 2B Top panel

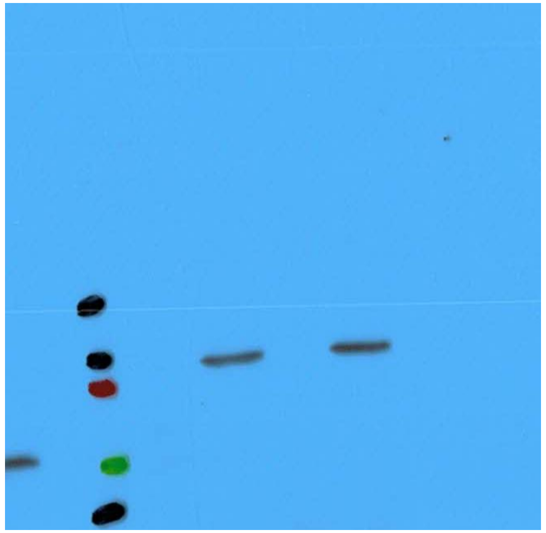

Figure 2B Middle panel

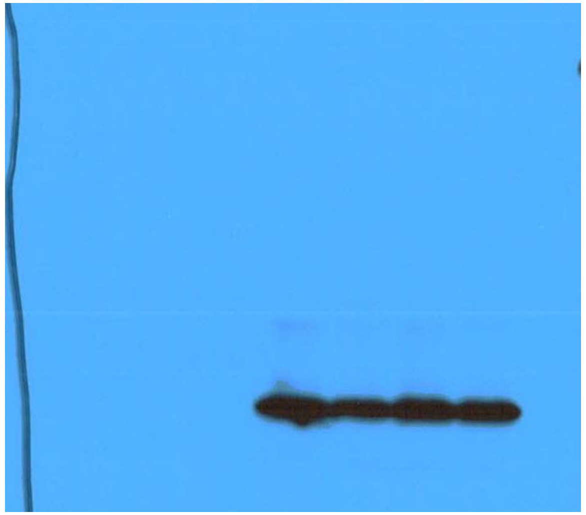

Figure 2B Bottom panel

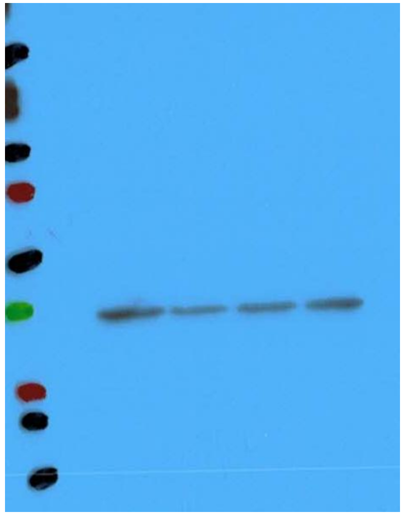

Note: The data in Figure 2B is from one of the repeated experiment.

Figure 3A Top panel

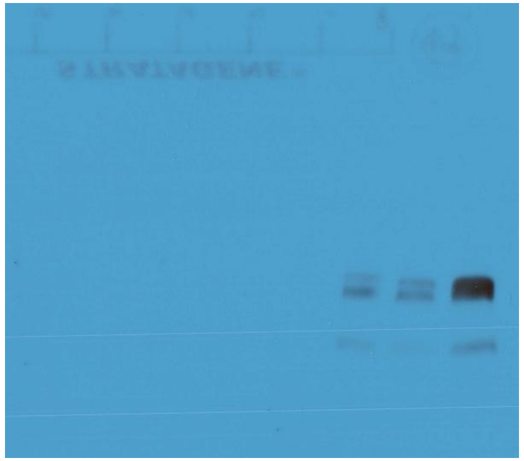

Figure 3A Bottom panel

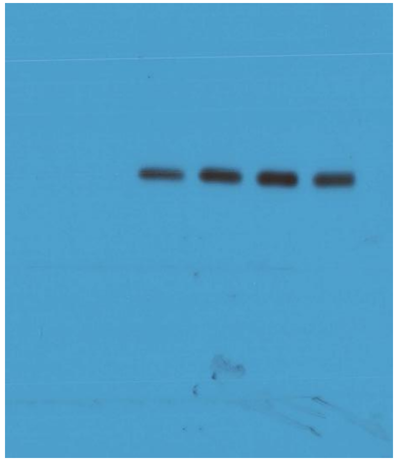

Figure 3B Top panel

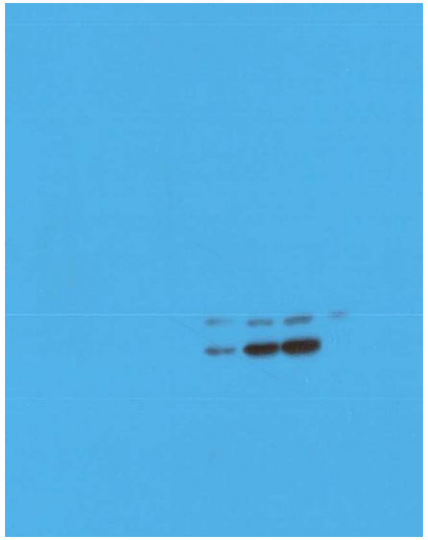

Figure 3B Middle panel

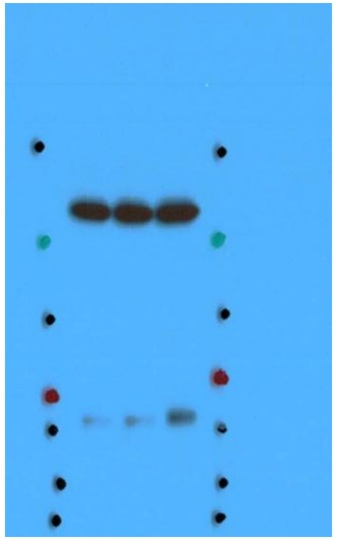

Figure 3B Bottom panel

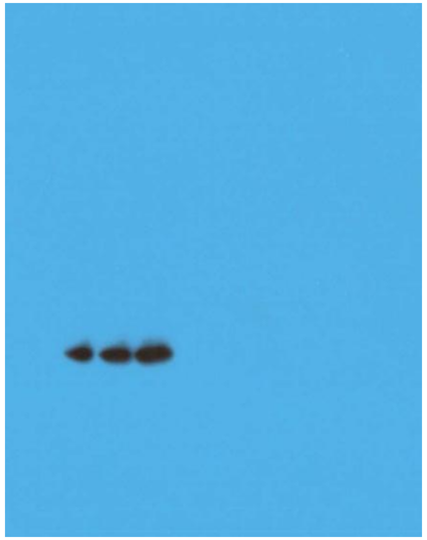

Figure 3C Top panel

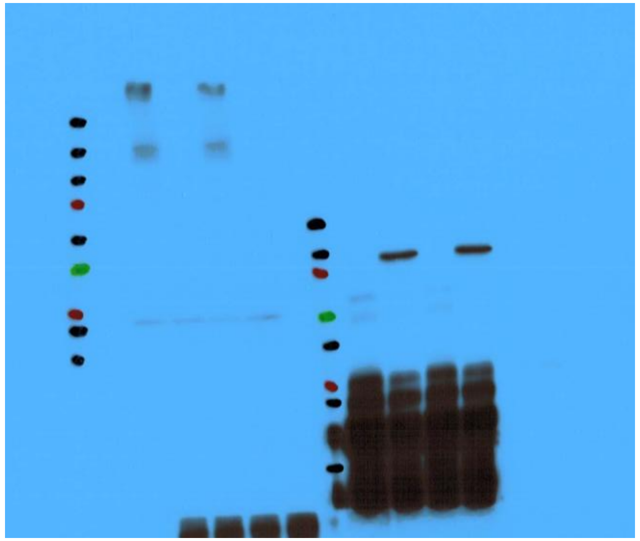

Figure 3A Bottom panel

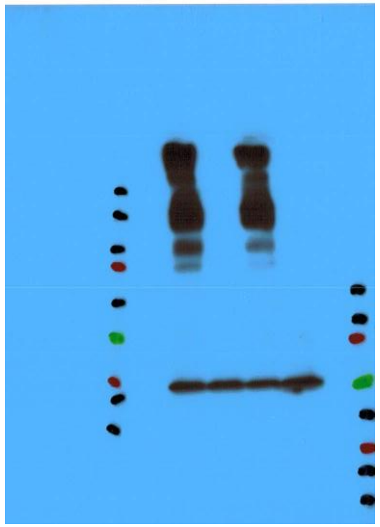

Figure 3D Top panel

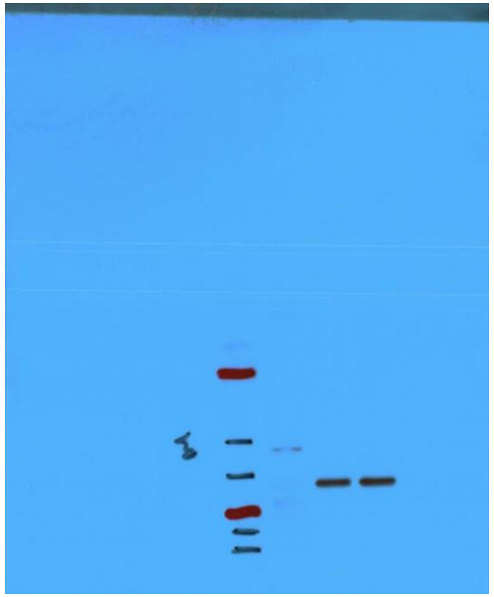

Figure 3D Bottom panel

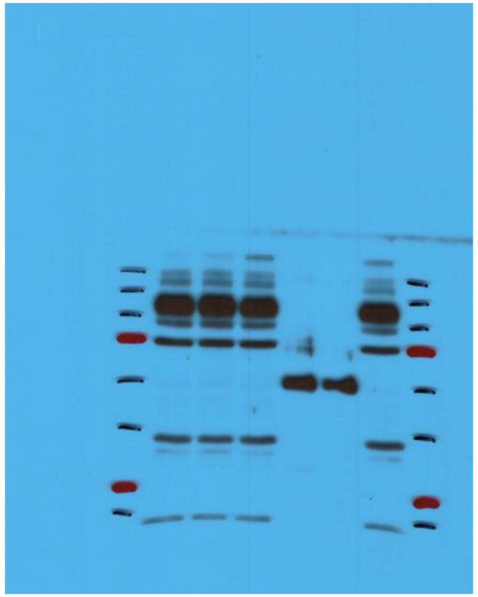

Supplement: Supplementary file 1 — Additional file 1 [file 13567_2025_1681_MOESM1_ESM.pdf]
